# The Experience of Key Stakeholders During the Implementation and Use of Trauma Therapy via Digital Health for Military, Veteran, and Public Safety Personnel: Qualitative Thematic Analysis

**DOI:** 10.2196/26369

**Published:** 2021-08-12

**Authors:** Lorraine Smith-MacDonald, Chelsea Jones, Phillip Sevigny, Allison White, Alexa Laidlaw, Melissa Voth, Cynthia Mikolas, Alexandra Heber, Andrew J Greenshaw, Suzette Brémault-Phillips

**Affiliations:** 1 Heroes in Mind, Advocacy and Research Consortium (HiMARC) Faculty of Rehabilitation Medicine University of Alberta Edmonton, AB Canada; 2 Department of Occupational Therapy Faculty of Rehabilitation Medicine University of Alberta Edmonton, AB Canada; 3 1 Field Ambulance Physical Rehabilitation Department Canadian Forces Health Services Department of National Defense Edmonton, AB Canada; 4 Leiden University Medical Centre Leiden University Leiden Netherlands; 5 Department of Educational Psychology Faculty of Education University of Alberta Edmonton, AB Canada; 6 Military Family Resource Centre Edmonton, AB Canada; 7 Health Professionals Division Veterans Affairs Canada Ottawa, ON Canada; 8 Department of Psychiatry University of Ottawa Ottawa, ON Canada; 9 Department of Psychiatry Faculty of Medicine and Dentistry University of Alberta Edmonton, AB Canada

**Keywords:** trauma, mental health, telemedicine, therapy, rehabilitation, digital health, psychotherapy, military, veteran, first responder, public safety personnel, teletherapy, psychotherapy, telepsychiatry, mobile phone

## Abstract

**Background:**

Exposure to occupational stressors and potentially psychologically traumatic events experienced by public safety personnel (eg, paramedics, police, fire, and correctional officers), military members, and veterans can lead to the development of posttraumatic stress injuries and other mental health disorders. Providing emergency services during COVID-19 has intensified the challenges. Owing to COVID-19 restrictions, mental health service providers offering support to these populations have had to rapidly pivot to use digital versus in-person methods of service delivery.

**Objective:**

This paper aims to explore the experience of mental health service providers regarding digital health service delivery, including the current state of digital mental health service delivery, barriers to and facilitators of the use of digital health for mental health service delivery experienced during the pandemic, and recommendations for implementing and integrating digital health into regular mental health service delivery.

**Methods:**

This embedded mixed-methods study included questionnaires and focus groups with key stakeholders (N=31) with knowledge and experience in providing mental health services. Data analysis included descriptive, quantitative, and qualitative thematic analyses.

**Results:**

The following three themes emerged: being forced into change, daring to deliver mental health services using digital health, and future possibilities offered by digital health. In each theme, participants’ responses reflected their perceptions of service providers, organizations, and clients. The findings offer considerations regarding for whom and at what point in treatment digital health delivery is appropriate; recommendations for training, support, resources, and guidelines for digitally delivering trauma therapy; and a better understanding of factors influencing mental health service providers’ perceptions and acceptance of digital health for mental health service delivery.

**Conclusions:**

The results indicate the implementation of digital health for mental health service delivery to military members, public safety personnel, and veterans. As the COVID-19 pandemic continues, remote service delivery methods for trauma therapy are urgently needed to support the well-being of those who have served and continue to serve.

## Introduction

### Background

The COVID-19 pandemic necessitated a sudden shift in the provision of mental health services from in-person to digital health delivery to comply with physical distancing restrictions [1]. This shift required adaptations at the organizational, service provider, and client levels. It was also experienced differently by the general population than by military members (MMs), veterans, and public safety personnel (PSP; eg, paramedics, police, fire, and correctional officers) dealing with symptoms and other effects of operational stress injuries (OSIs). As a result, continuing access to mental health supports for trauma-affected populations, including MMs, veterans, and PSP, was pivoted to digitally delivered trauma therapy.

In addition, MMs and PSP are critical to the COVID-19 pandemic response and management as they respond to medical emergencies, enforce public health orders, and support infection control and prevention initiatives [2]. Although accustomed to performing in complex, ambiguous, high stake, and rapidly changing environments, the context of the COVID-19 pandemic has amplified these factors and led to operational environments that are highly stressful and uncertain [3]. Therefore, supporting MMs and PSP in a timely and responsive manner is critical, particularly as increased occupational and operational stressors are likely to continue during and beyond the COVID-19 pandemic and can lead to OSIs.

### Digital Health for MMs, Veterans, and PSP

Creative approaches to mental health service delivery that maximize access to services while minimizing physical contact and proximity are required as a result of the pandemic. Digital health is an umbrella term that encompasses a variety of health technologies such as teletherapy, telemedicine, eHealth, mobile health, and health information technology delivered through various modes such as videoconferencing or telephone and has offered a means by which to address immediate mental health needs. For MMs, veterans, and PSP living in rural or remote areas, digital health can facilitate access to mental health treatment by removing barriers to access associated with transportation or service availability [4]. The ability to access mental health treatment from the comfort and safety of a home environment has been identified as a facilitator for individuals experiencing social anxiety or with concerns regarding privacy and stigma [5-7].

Before the COVID-19 pandemic, the adoption and implementation of telehealth as a means of mental health service provision was slow and difficult to sustain [8], and the response of mental health clinicians to the shift to or adoption of digital health to provide care for MMs, veterans, and PSP was mixed [9]. Barriers to widespread and sustained digital health use were associated with concerns regarding the equivalency of telehealth-delivered services to face-to-face treatment, establishment of a therapeutic alliance, client and service provider acceptance of digital health, technical connectivity challenges, patient privacy and confidentiality, software and equipment availability, usability and reliability, associated costs, and regulatory concerns [1,9-12]. Recommendations associated with digital delivery, in general, emphasize clinician training and supervision around the use of technologies and mental health interventions [13]; development of guidelines and policies for software, web-based platforms; and patient consent [14].

Regulatory colleges for health care professionals have provided some practice standards and guidelines for the use of digital health many of which have been developed specifically in response to COVID-19. Regulatory colleges have offered initial standards and guidance regarding privacy and confidentiality, consent, risk management and safety, technical issues and security of data, ongoing training regarding both professional and technical competency, and possible insurance or jurisdiction issues [15-18]. However, given the rapidly changing landscape, little research has been conducted exploring how these standards have been taken up by frontline service providers and factors that facilitate their implementation. In particular, issues relating to privacy are especially salient given not only the widespread stigma associated with mental health concerns in the military or PSP organizations but also the sensitivity of disclosures that may occur when working with MMs or PSP who have high security clearance.

Despite these barriers, encouraging evidence supporting the use of digital health was identified through a systematic scoping review conducted by members of this research team in advance of this study regarding digital health delivery of trauma therapies for MMs, veterans, and PSP [19]. This review illustrated that the delivery of cognitive processing therapy (CPT), prolonged exposure therapy, and behavioral activation therapy using videoconferencing demonstrated comparable effectiveness to in-person treatment in reducing posttraumatic stress disorder symptoms in MMs and veterans [19]. This scoping review identified significant knowledge gaps. Specifically, the majority of available evidence comes from studies conducted in the United States with active-duty MMs and veterans where service providers predominately used CPT. Far less research has been conducted on other treatment modalities or on the treatment of PSP; practically no research on digital health for MMs, veterans, and PSP has been undertaken in the Canadian context. It is imperative that these knowledge gaps be addressed to promote continued, effective, and ethical use of digital health technologies during and beyond the pandemic.

### Purpose

The purpose of this study is to explore the perspectives of mental health service providers regarding the shift to using digital health to facilitate mental health service provision for MMs, veterans, and PSP as a result of the COVID-19 pandemic. Specific study objectives include the determination of the following:

- The current state of digital health use in mental health service delivery for the target populations.
- Barriers and facilitators experienced by mental health service providers in the transition to the use of digital health.
- Recommendations for the implementation and integration of digital health into regular mental health service delivery by mental health service providers.

## Methods

### Overview

This study used an embedded mixed-methods design [20] in a community-engaged research setting [21]. The primary method was qualitative and involved focus groups with key stakeholders who had direct knowledge and experience, as well as the most useful and timely information sensitive to the context of the research population and question [22]. Engagement with key stakeholders aimed to explore the history of events, the shift to digital health use, what occurred with the shift to the use of digital health (eg, changes in service delivery, access, practice, policy, and technology use), what needs, problems, and solutions have arisen (eg, digital health approaches), and important considerations going forward [23-25]. Quantitative descriptive data were also collected and nested within the thematically analyzed qualitative data. Ethical approval was obtained from the research ethics board of the University of Alberta before study initiation.

### Participants

Study participants included representatives from the following groups: (1) MMs, PSP, and veterans working in peer-support, health or wellness, or mental health service positions; (2) multidisciplinary mental health service providers; (3) organizational leaders and policy and decision-makers from local, provincial, and federal PSP, military, and veteran organizations; and (4) subject matter experts and researchers (eg, in digital health, mental health, technology, privacy, security, and implementation). Although attempts were made to recruit MMs, PSP, and veterans who could primarily be categorized as having lived through the experience of a posttraumatic stress injury or OSIs and who were directly receiving digital health trauma therapy, no participants agreed to take part in the study. For MMs and PSP, this was likely because of deployments and frontline service provision during COVID-19.

### Inclusion and Exclusion Criteria

Participants were included and excluded based on the criteria described in Textbox 1.

**Inclusion and exclusion criteria of the participants.**

**Inclusion Criteria**

- If they had population-, service provision–, subject-, or organization-specific expertise
- If they were able to offer insights into real-world service delivery, practice, policy, or technology; privacy and security issues in the Canadian context; practical considerations and experiences associated with the use of digital health for mental health service delivery; or realistic solutions associated with digital health service delivery to military members, public safety personnel, and veterans in Canada
- If they were English speaking
- If they were able to provide informed written consent

**Exclusion Criteria**

- If they were unfamiliar with the population or use of digital health to deliver mental health services
- If they were unable to contribute meaningfully to an exploration of the use of digital health
- If they were non–English speaking
- If they were unable to provide informed consent

### Recruitment

Study participants were recruited through word of mouth and convenience, snowball, and purposeful sampling. Key individuals in military, veteran, and PSP organizations were contacted by phone, and emails were disseminated through relevant networks and organizations with local, provincial, and national reach. Key stakeholders (or their designates) were asked to contact the research team directly and were screened for inclusion. Anonymity and confidentiality were reviewed, and consent was obtained before the commencement of study participation.

### Data Collection

Data were collected between August and October 2020. Quantitative data were collected using questionnaires that captured demographic information (eg, age, gender, profession, organizational affiliation, years of service, and province of residence), participants’ comfort level with particular digital health platforms and technology, and their perceptions of facilitators of and barriers to digital health service delivery. Questionnaires were administered on the web via REDCap (Research Electronic Data Capture)—a secure, web-based software platform hosted at the University of Alberta and designed to support data capture for research studies [25]. Qualitative data were then collected through 90-minute focus groups (n=5; 3-6 participants per focus group) and interviews (n=1) conducted and recorded over the phone or encrypted Zoom (Zoom Video Communication Inc) software [26]. Examples of focus groups and interview questions are given in Multimedia Appendix 1. Each focus group was purposely heterogeneous, with respect to professional representation and experience with digital health, to allow for broad cross-talk and pollination of complementary and alternative ideas, experiences, and conversation, resulting in rich comprehensive data. The focus groups and interviews were guided and facilitated by senior members of the research team. Key topics of discussion included the previous and current state of using digital health for mental health service delivery in the midst of COVID-19 pandemic; barriers to, facilitators of, and recommendations for the use of digital health technologies to deliver mental health services to MMs, veterans, and PSP; digital health technological issues, acceptance, and methods of delivery; clinical effectiveness; and needs, including infrastructure and implementation.

### Data Analysis

Qualitative and quantitative data were analyzed using standard analytical procedures. Quantitative data were analyzed descriptively and statistically using SPSS (IBM Corp). Audio- or video-recorded focus groups and interviews were transcribed and thematically analyzed [27], both deductively and inductively, following an iterative process. Deductively, initial codes were developed based on focus group topics and study objectives. Inductive coding involved identifying themes that emerged from the data [28]. In total, 3 junior researchers independently conducted open coding for each focus group, after which a senior researcher reviewed and refined the codes. These were then combined and tabulated into preliminary themes. The analysis of the preliminary themes by the collective research team followed, with differences resolved through discussion. A proposed thematic theory underwent collective analysis; the preliminary themes were modified; key quotes were isolated to illustrate the selected themes; and the final thematic narrative was prepared.

## Results

### Questionnaire Results

The overall focus of this analysis was to explore the experience of mental health service providers working with MMs, veterans, and PSP during the transition from in-person to digital health service delivery of mental health interventions. Demographic details of the study participants who completed the digital health questionnaires (N=31) are shown in Tables 1 and 2. Study participants had diverse professional backgrounds, resided in various regions of Canada, and had varying levels of experience within their respective professions and with digital health.

**Table 1:** Occupational and geographical characteristics of the study participants (N=31).

Characteristics		Values, n (%)
**Organizational affiliation (n=31)**		
	Alberta Health Services	4 (16)
	Canadian Armed Forces or Department of National Defense	5 (20)
	Postsecondary institution	4 (16)
	Municipal Police Service	2 (8)
	Operational Stress Injury Clinic	2 (8)
	Canadian Coast Guard	3 (12)
	Private Mental Health Clinic	3 (12)
	The Royal Canadian Legion	4 (16)
	Other	4 (16)
**Profession (n=27)**		
	Paramedic	4 (16)
	Psychiatrist	3 (12)
	Police officer	2 (8)
	Administrator	4 (16)
	Psychologist	4 (16)
	Physician	2 (8)
	Chaplain	2 (8)
	Researcher	3 (12)
	Safety advisor	1 (4)
	Other allied health professional	2 (8)
**Province (n=24)**		
	British Columbia	3 (12)
	Alberta	15 (62)
	Ontario	3 (12)
	Quebec	1 (4)
	Nova Scotia	1 (4)
	Saskatchewan	1 (4)
**Years of service (n=28)**		
	1-4	1 (4)
	5-9	4 (16)
	10-14	3 (12)
	15-19	8 (24)
	20-24	8 (24)
	25-29	2 (8)
	30-34	1 (4)
	≥35	1 (4)

**Table 2:** Age and gender of the study participants.

Characteristics			Values, n (%)
**Age (years; n=23)**			
	18-34	0 (0)	
	35-49	14 (60.9)	
	50-64	9 (39.1)	
	≥65	0 (0)	
**Gender (n=24)**			
	Male	13 (54.2)	
	Female	11 (45.8)	

A variety of means of communication used to facilitate mental health service delivery were identified by participants, including videoconferencing platforms, email, telephone, and text. Table 3 shows the digital health methods that participants felt comfortable using.

**Figure 1:**
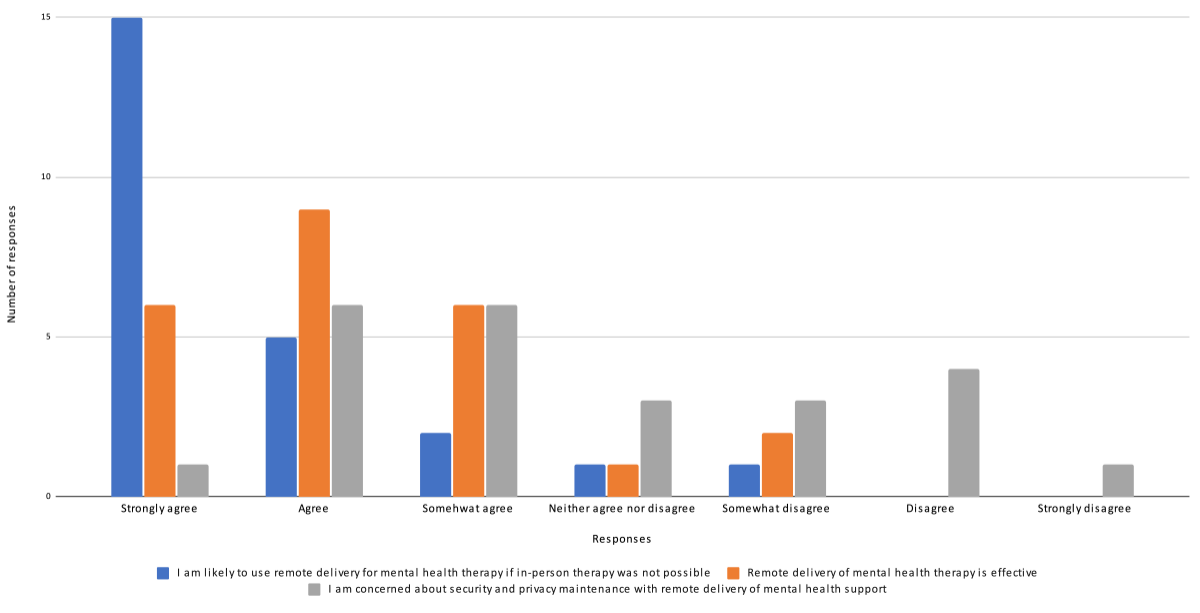
Participants’ perceptions of intention to use digital health, effectiveness, and privacy and security risk (N=24).

Intention to use remote delivery, perceptions of the effectiveness of digital health for mental health service delivery, and concerns about privacy and security were rated using a 7-point Likert scale (Figure 1). The vast majority of participants somewhat agreed, agreed, or strongly agreed that they were likely to use digital health delivery of mental health services if in-person therapy was not available. Most respondents also perceived that mental health services delivered remotely were effective. Over half of the respondents expressed at least some concern regarding the maintenance of security and privacy when delivering mental health services through digital health means. Multiple facilitators of and barriers to digital health delivery of mental health services were also identified by participants in the pre–focus group questionnaires (Tables 4 and 5). Common barriers included lack of stable, interruption-free internet access, lack of personal presence, and challenges in developing a therapeutic relationship, whereas identified facilitators included decreased perception of stigma as well as greater convenience and access to mental health services. Consideration of the abovementioned factors associated with the delivery of trauma-focused therapies to MMs, veterans, and PSP before engagement in focus groups and interviews set the context for interprofessional discussions around using digital health for mental health service delivery.

**Table 3:** Technology and platforms participants have used and feel comfortable using for digital health (N=31).

Technology and platforms	Values, n (%)
Email—personal	12 (11)
Email—work	11 (10)
Phone—personal	15 (13)
Phone—work	11 (10)
Text—personal	9 (8)
Text—work	4 (4)
Teleconference without video	10 (9)
Google Meets	5 (5)
GoTo Meeting	0 (0)
Cisco Webex	5 (5)
Lifesize	0 (0)
Zoom Business or Enterprise	4 (4)
Zoom Health Care	10 (9)
Zoom	12 (11)
Other^a^	4 (4)

^a^*Other* platforms reported included Skype for Business (n=1), Microsoft Teams (n=2), and FaceTime (n=1).

**Table 4:** Facilitators of digital health delivery (N=24).

What do you think some of the benefits of remote delivery of mental health support are?	Values, n (%)
Not having to travel	22 (23)
Greater convenience	21 (22)
Greater availability of services (ie, more therapists available)	19 (20)
Increased privacy	14 (15)
Reduced mental health stigma from others	17 (18)
Other	3 (3)

**Table 5:** Barriers to digital health delivery (N=24).

What do you think some of the challenges to remote delivery would be?	Values, n (%)
Lack of appropriate internet access	22 (21)
Reduced privacy	11 (10)
Reduced security	8 (8)
Interruption to communications	19 (18)
Lack of personal presence	17 (16)
Difficulties developing a therapeutic relationship	14 (13)
Challenges delivering or receiving therapeutic modality	14 (13)
Issues of safety in case of negative response	1 (1)

### Qualitative Results

Thematic analysis of the qualitative data isolated three main themes: (1) being forced into change, (2) daring to deliver mental health services using digital health, and (3) future possibilities offered by digital health. In each theme, participants’ responses encompassed considerations for service providers, organizations, and clients. What followed was an elaboration of these themes, together with tables of subthemes and supporting quotes.

#### Theme 1: Forced Into Change

##### Service Providers

Providers’ willingness to use digital health was integral to delivering trauma therapy to MMs, veterans, and PSP amid physical distancing restrictions (Textbox 2). Personal experience, skill level, and readiness to adopt technology were reasons for both mental health service provider excitement around and reluctance to accept the use of digital health to deliver trauma therapy.

**Forced into change—service provider subthemes.**

**Addressing Personal Attitudes and Mindset**

- *I think we’re the bigger problem then patients are in some cases. I think we’re the larger barrier to being able to shift to a digital-based solution. Because I was concerned about it, I was anxious.* [Focus group 3; P18]
- *But it was provider willingness, I think some of it was technology-based, some of it was just the unwillingness of certain providers and I don’t know why - was it you’re missing something? was it them - how am I licensed to do this? Some college issues? Personal professional issues? Or maybe it’s just fear of the unknown - ‘well I don’t understand this technology so I’m just going to stick with pen and paper.’* [Interview, P1]

**Managing Increased Workflow Demands**

- *It definitely was a transition for us on a clinician level. There is more to manage, there’s more things to review. So I think for some people it kind of slowed down the process because we needed to [...] talk about what it meant to do these virtual sessions, [...] what it meant to [...] find a place in your home where you can do this trauma work that’s going to be sensitive, that’s going to be appropriate, [...] the logistics.* [Focus group 3; P14]

**Accessing Client Information**

- *[F]or somebody with a head injury, [...] they can't get a neuro psych assessment; that's just been put on hold indefinitely. Again, you know, not being able to get this kind of multidisciplinary information to inform the supports that I'm giving or the treatment direction that I’m giving you know what part is post-concussion syndrome and what part is mental health and how am I going to tease that out? Well, I don't have the information. So we are kind of making do.* [Focus group 5; P33]

**Exchanging Therapeutic Material With Clients**

- *[H]ow do we get the worksheets to the person? How do we get the person the materials that are part of the treatment? How do we have a conversation where I can’t see the worksheets? For example, sending emails - so I have to remember I got to make sure that I send the email for this week’s homework or I gotta remember on my end to make sure that I send out the workbook for the person to do cognitive processing therapy, and then it might take a week or two for it to actually get to them, given how the mail system is right now.* [Focus group 3; P14]

**Making Reliable Clinical Observations: The Body Tells the Story**

- *We have been trained our whole careers [...] to vet all emotion out of everything we write, every report has to be completely neutral. [...] [I]f we don’t have the body to tell us the story, if we don’t have that ability to observe, we’re really limited [...]. Tone of voice and all those things are really helpful, but more seasoned Cops can become really good at [...] hiding all real emotion.* [Focus group 2; P9]

**Facilitating the Therapeutic Alliance**

- *[T]he physical connection and the closeness of actually being with someone is a huge part to allowing them to trust you, to feel your empathy, to allow you into what they’re going through, and doing it screen to screen, especially when there are glitches and stuff that happens, just is not as effective and I have noticed that our members are not on board with it.* [Focus group 2; P13]
- *From my point of view why it doesn’t work again, is just that there’s something real with the energy you share with someone when you’re with them. And this takes that away. So from my point of view it takes so much longer to build trust, to get people to open up. So I think from my point of view that’s what I saw, they were just not as willing to be open and honest of what they’re going through... they’re just not as comfortable having that conversation online versus face to face. And I understand that, because I really feel like that connection is not there... members do not want to have these meetings online, they just don’t.* [Focus group 2; P13]

**Conducting Risk Assessments and Safety Planning**

- *I would find it very difficult to do [...] a real thorough assessment if someone was actively suicidal in a virtual setting. [...] I would find it very difficult to see how that can be conducted wholesomely, also without seeing – because agitation can be seen in the feet. There’s so much that I would take under consideration when I assess a patient who is suicidal.* [Focus group 1; P3]
- *Where is your client geographically? If you lose contact with them and they’re in crisis how are you going to get them help? So really thinking through, what are the clinician guidelines about knowing where people are?* [Focus group 5; P33]

Workload responsibilities reportedly increased with the shift to remote delivery of mental health service provision. The proficient use of technologies was challenging for some and resulted in increased work time and administrative duties. Providing clients with therapy resources or exchanging confidential materials required creative problem-solving. One participant described needing to be a clinical service *technology wizard* since the start of the COVID-19 pandemic. Participants also noted that the additional time and effort required to implement practice change was often underestimated or ignored by employers.

Communication with colleagues around client care was impacted by a shift to digital health. Participants described having fewer opportunities to confer and collaborate with their colleagues. They also indicated that client information was not as readily accessible, thereby making assessments and case histories more difficult to complete. Furthermore, securely and remotely accessing and transferring client files required additional consideration.

The delivery of trauma therapy also requires careful adjustment and adaptation. Participants noted that the sense of engagement and energy were notably different between in-person and digital health sessions. They also noted that assessing and monitoring nonverbal cues in a virtual setting is challenging. As MMs, veterans, and PSP typically suppress emotions, Mental health service providers rely on nonverbal facial expressions and body language to determine what a client may be feeling. One participant noted that “the body is what tells us the story.” The inability to “see below a client’s head and neck” can impede an SP’s ability to pick up on mental health indicators (eg, overall appearance, hygiene, and psychomotor agitation). Participants also felt that digital health negatively impacted the therapeutic alliance and, at times, resulted in the clinician feeling distant or cut off from their client.

Assessing risk over digital health was also consistently identified as a topic requiring consideration. Participants questioned how to best manage client disclosures of suicidal ideation, intent of harm to self or others, or domestic violence. Dealing with such situations was identified as the primary reason that using digital health was uncomfortable and anxiety provoking.

##### Organizations

Although organizations and service providers wanted to provide the best mental health services possible to MMs, veterans, and PSP with OSIs during the COVID-19 pandemic, they encountered numerous barriers to service provision (Textbox 3).

Organizations and regulatory bodies for regulated health professionals were unprepared for a sudden shift to digital health service delivery. For example, some participants noted that adopting digital health was prohibited by organizational mandates just days before the COVID-19 lockdown. As a result, policies, practices, systems, and resources were viewed as being limited or inappropriate. Mental health service providers indicated that these realities impeded their use of digital health when responding to client needs.

Security and privacy concerns were strongly highlighted. Participants noted that access to organization-specific computer hardware, software, and systems was lacking, making it necessary for them to use personal devices in both work and home environments. Given this reality, organizational security and privacy policies were often either overly restrictive or not restrictive enough to address the unique factors associated with offering trauma therapy from home.

**Forced into change—organization subthemes.**

**Changing Organizational Practices**

- *Some agencies are so stuck in the way they’ve always done it. (...)[S]o this is a whole new chapter, truly.* [Focus group 2; P9]
- *We don’t have the latitude in the military on our defense wide network of deviating from the rules, because everything is sort of being scrutinized, and so we’ve got clinicians who want to be able to communicate because the patients are asking to communicate on email. [...] [T]rying to find a way to do it within the bounds of the rules has been a challenge for us.* [Focus group 3; P21]

**Exploring Digital Health Platforms to Meet Client Needs**

- *[W]hile everyone is keen to use Zoom, [...] we’re not able to use it on our network because of the concerns over security. So it raises issues for us in terms of what platforms we can actually use on our network; [...] the military but also to our federal partners [...] are bound by the same security parameters so that’s a real impediment for us.* [Focus group 3; P21]
- *[T]he policies that have been in place and the privacy concerns that have existed since we started have been really huge barriers to our delivery of care. Our population usually has cognitive deficits, has difficulty with technology and just problem solving at its most basic. For us, one of the policies for example is to encrypt all emails. People could not figure out how to unencrypt them and access our Zoom groups or our Zoom meetings if we encrypted them.* [Focus group 1; P5]

**Managing Security Concerns**

- *I agree that to just start up with any platform and start talking to patients without any real understanding of the privacy implications is a real risk.* [Focus group 1; P3]
- *Videotaping that has always been something that is part of our sessions for therapy purposes. That has been turned off by [agency] as a status-quo for all our sessions on Zoom, so even when we went to leadership to turn that off so we could Zoom videotape, and the patient gave consent, we couldn’t do that because the legal team wouldn’t allow us.* [Focus group 1; P5]

##### Clients

The service provider participants thoughtfully considered ways to enable clients to access mental health services and overcome barriers to digital health use (Textbox 4). These participants spoke of the importance of ensuring that clients have the appropriate technology to support digital health delivery, understand how to use the technology and appreciate the risks and benefits of using digital health, and provide consent before engaging in trauma therapy. Participants highlighted that digital health also presumes a certain level of socioeconomic prosperity (ie, access to a computer, high-speed internet, and a private or secure location), which may not be attainable for all clients. As some clients may be uncomfortable with the possibility of a session being monitored by an employer or third party, study participants emphasized the importance of reassuring clients of the confidential nature of therapy sessions.

**Forced into change—client subthemes.**

**Managing Inequalities**

- *If I’m out of work and or don't have [...] high speed internet, and I only have a phone or [...] a tablet that’s maybe outdated, [...] my experience engaging with you as a provider is going to be very different [...] interrupted or lower quality or I’m going to be so preoccupied. [...] [T]hat’s really a socioeconomic issue.* [Interview; P1]
- *Immediately there were some challenges with some of our clients, knowing how to use technology being able to navigate the digital world, particularly for older clients and then the technology itself whether that was the specific one that we were using or other ones. [...] Other issues were related to people not having appropriate technologies.* [Focus group 5; P29]

**Addressing Confidentiality and Privacy Concerns**

- *[Y]ou actually have to tell clients that they shouldn't take the call sitting against an airplane hangar while there's people passing in front of it. That they actually need some privacy and security in a place where they won't be interrupted in order to do this work. So because we're not containing the space, we need to educate our clients about how to create that space for themselves.* [Focus group 5; P33]
- *[I]n terms of privacy, if you’re now consulting by video call [...], you could potentially see their background and you could sort of see into their homes, [...] people walking by or dogs or cats or children.* [Focus group 1; P4]
- *I have most of my conversations as conversations because they don’t want anything in writing ever. [...] [A]nything that’s important they want to know it’s staying between you and them.* [Focus group 2; P11]

**Building Digital Relationships**

- *For me, my experience is when I'm working with folks like that, if I have a relationship with them already talking on the phone is, is really effective. We don't do video conferencing. But if I don't have a relationship with them and then I want to phone and talk to them about an issue, it's not as effective.* [Focus group 4; P23]
- *[T]hey have so many trust issues, and they have anxiety about going in to see a clinician in the first place that, now you throw the computer in there, they’re unsure of what’s being recorded, [...] who has access to it [...]. I think it just increases their anxiety about opening up and sharing.* [Focus group 2; P13]

**Facilitating Coregulation**

- *It’s that self-soothing piece because you’re not gonna be in the room to be able to support me. [...] [I]t’s really giving people the tools [...]. They’re being activated in a way that you can’t necessarily see, but also could stay activated afterwards [...]. [T]hey might process that more quickly if they’re in the room with you.* [Focus group 4; P23]
- *[C]ops are reluctant to talk about emotions at all; [...] they don’t want to take their cork out of the bottle if there’s not somebody to catch them.* [Focus group 2; P11]

**Accommodating for Cognitive and Trauma Challenges**

- *[W]ith members who have been dealing with PTSD [posttraumatic stress disorder], just practically, sitting and staring at a screen for an hour, would be very difficult [...]. Bright lights. Some guys can’t even watch TV, so I think even practically that would be a big thing.* [Focus group 2; P13]
- *There are absolutely people who have a huge adversity to technology, and it’s now linked to their trauma, [...] [T]hey’re hearing it and are helpless to fix the situation.* [Focus group 2; P9]
- *[S]ome members, [...] although they feel it’s not going to be as productive as face to face, would still do it; [other] members would just be like, “no thanks, I would rather just deal with it.” [...] [I]t would be case to case.* [Focus group 2; P13]

Conducting trauma therapy using digital health, although workable for many clients, was not viewed by the participants as appropriate for all MMs, veterans, and PSP. Some participants felt it may be more challenging to build therapeutic alliances virtually rather than in person with certain clients. Participants emphasized that care must be taken in selecting the best format of service delivery for each individual client, especially MMs, veterans, and PSP, who may experience issues of trust and attachment.

Participants expressed that clients with cognitive dysfunction, who have experienced a brain injury, and who are highly emotionally dysregulated and in frequent need of active coregulation may not be suitable for digital health delivery of trauma therapy. Furthermore, those clients for whom technology is a trigger (eg, police officers who may be required to watch graphic web-based content) may also not benefit from digital health-delivered trauma therapies.

#### Theme 2: Daring to Deliver

##### Service Providers

The use of digital health afforded service providers unexpected benefits (Textbox 5). Most participants noted that digital health service delivery *saved time* because of a lack of commuting and fewer workday interruptions, which enabled them to respond to more clients. Participants noted that the efficiency experienced was also impacted by changes in their attitudes toward digital health and by gaining familiarity with the technology and software platforms.

Digital health offered new intervention possibilities and options for providing outcome-oriented services. Some mental health service providers reported adapting psychoeducational approaches, developing new ways of conducting exposure-based interventions, and facilitating client connections to support groups. Participants noted the importance of allowing service providers to develop creative solutions, with 1 participant stating, “Stay nimble...keep looking at how to make things better...leverage this.” Furthermore, as service providers acclimated to digital health service delivery, they were better able to accommodate for the lack of access to nonverbal cues. Provision of therapy to a client in their home environment afforded service providers a glimpse into the client’s living situation, offering valuable therapeutic information.

**Daring to deliver—service providers.**

**Increasing Efficiency Through the Use of Digital Health**

- *Pre-COVID there were lots of barriers to virtual care and [...] working from home, but we have proven during this crisis that it can be done and we can be as efficient or almost as efficient working from home and taking care of our patient than we would be sitting in our offices with them in front of us.* [Focus group 3; P17]
- *[I]t became, I guess, time management wise, actually a lot easier. She could sit at our console, get a video screen, [...] and do a team's call with the one, the two or even a group session.* [Focus group 4; P25]
- *The fact that I don’t have walk-ins has allowed me to do a lot more with more patients. That I’m not interrupted by people knocking on my door, I can finish on time, I can focus better in my sessions, it’s easier, and I can still have patients communicate with me through my secretary so that I can get back to them when I have time. Putting that barrier actually helped me focus more on my work.* [Focus group 1; P5]

**Accepting and Gaining Familiarity of Digital Health**

- *There’s been this real kind of shift in my thinking from ‘this is second rate. This is not as good as the in person’ to really try to dig in and find what's the unique benefit of a video platform in terms of if we’re going to be doing this for a while, how do we lean into the technology and what it gives in terms of immediacy, of consolidation and implementation?* [Focus group 5; P33]
- *I’ll FaceTime somebody now instead of phoning them because we’ve gotten used to looking at people on screens and so I feel that that's actually been a third benefit for us here is the use of FaceTime, as opposed to just a phone call. So you can actually read some body language and although it's through a camera, look at people in the eye.* [Focus group 4; P22]

**Creatively Delivering Interventions**

- *So for me I was still in the office, facilitating exposures, where I could. For some folks, though, that I was working with that were immuno-compromised. Well, we couldn't do face to face live exposures and so we took it online. And so what that looked like was we started with the members sending me a list of videos that she needed me to record on my phone [...] and I walked around headquarters with my phone trying to be as steady as I could and recorded them, [...] So those were really, really successful and she was really happy with them and there was really good feedback.* [Focus group 4; P22]

**Enhancing Insight Into a Client’s Life Circumstances**

- *[Digital health provides] a glimpse into their world. You see what their environments look like; [...] whether they're in a state of chaos, just based on their room or the noise in the background. So some of those little things give you a bit of a sense of where they're at and what's going on for them. So I think that is [...] beneficial.* [Focus group 5; P29]

##### Organizations

Adaptations at the organizational level were made to enhance digital health service delivery (Textbox 6). Participants reported that organizations that seemed to be able to adapt to digital health most effectively embraced new opportunities for resource sharing and interagency collaboration. For example, connections with colleagues and national and international experts were also made more possible as a result of digital health. The shift to digital health also enabled organizations to expand their service offerings, build greater capacity, and facilitate more equitable mental health service provision, particularly with geographic limitations being removed by digital health. This move to more equitable mental health service provision was deemed to be extremely beneficial and relevant to MMs and PSP who are often required to work in remote and isolated locations.

**Daring to deliver—organizations.**

**Sharing Agency Resources**

- *[Digital health has been] positive in terms of helping other agencies create care options, [...] start reintegration programs. Zoom has been tremendous at connecting agencies. [...] I have done a lot of teaching over Zoom since all of this has started. So, that in terms of teaching [...] or connecting agencies [...] that has been awesome! [...] [I]t’s one level removed from the care to the individual, but I think it’s still so relevant.* [Focus group 2; P9]

**Providing More Equitable Services**

- *The reality of that lived experience is the occupational injury clinics and the therapeutic resources are concentrated in the metropolitan environments. So, prior to COVID, we took a stance that the support we were able to provide was equitable but not equal. [...] [A]n unintended benefit is that the playing field is now level in that everybody has the exact same type of interaction.* [Focus group 1; P2]

##### Clients

The effectiveness of digital health for use with MMs, veterans, and PSP was associated with several factors (Textbox 7). Participants identified the benefit of proactively reaching out to clients to provide regular support and information regarding how and when they might be able to access digital health therapy.

Overall, the participants perceived that the use of digital health reduced barriers to access and enhanced client comfort during appointments. The participants reported that the no-show rates for client sessions were unchanged in the shift to digital health and that missed appointments decreased. The strength of the therapeutic relationship impacted the ease with which service providers were able to transition from in-person to digital health delivery, with transitions being easier when a therapeutic relationship had been established through in-person sessions. Participants perceived that clients demonstrated a *forbearance flexibility* with the shift to digital health and an appreciation for the steps taken to maintain their safety and mental health during the pandemic.

**Daring to deliver—clients.**

**Continuing Access to Appointments**

- *I think for trauma-focused treatments my no-shows have gone down.* [Focus group 3; P14]
- *Very often now people are in their own space, they’re comfortable, there's actually more intimacy and there can be more safety.* [Focus group 5; P33]
- *[W]e have not seen fewer clients or had fewer sessions. They’re very comparable. And we have not seen any change in our no-show rate for appointments, [...] So clients are attending, [...] [and] getting just as many sessions.* [Focus group 3; P15]

**Creating Connections**

- *My colleague and I ended up doing some groups, partly so people felt connected, and yet we were teaching skills and mindfulness [...]. So I think that there’s a real benefit to that, even just to increase access.* [Focus group 3; P16]
- *[We’re] really staying in close contact. Right from that first week, clinicians [...] phoned every client to say ‘we know this is a difficult time, [...], here are the options available to you.’ So that proactive outreach, I think, was a really important piece of keeping our clients engaged.* [Focus group 3; P15]
- *We started a sentinel program in the unit so we had people responsible to check in on their people routinely.* [Focus group 1; P6]

**Adapting to Use of Digital Health for Mental Health Service Delivery**

- *[W]hereas it took longer to establish a therapeutic relationship at the beginning of COVID, I'm finding that [...] it’s pretty seamless now. So people are adapting. I'm adapting. People are adapting.* [Focus group 5; P33]
- *The feedback I’m getting from the ships is that yeah, this is different, but what we’re providing is good and sufficient. I mean they understand the constraints, they very much appreciate that we’re not putting them at physical risk by insisting that we send a team in. And so there’s that patience, forbearance flexibility that’s required on the other end to make this work. But our observations is that the folks are A: professional enough to deal with that, but B: they realize the need for the support. So they take the compromised approach rather than just say, don’t bother. So there’s an adaptation that's occurred on everybody’s part right now, and it’s not just technology.* [Focus group 4; P25]

#### Theme 3: Looking Forward

##### Service Providers

Viewing current circumstances as a catalyst for change, participants made recommendations to enhance mental health service delivery based on their experience of digital health use (Textbox 8). Suitability of clients for trauma therapy using digital health was discussed, with safety concerns and risk factors being primary considerations. Participants noted that using digital health to deliver mental health services could work very well for clients experiencing anxiety, depression, or fear of stigma or finding it difficult to get to mental health appointments. However, they also noted that not all of their clients fared well using digital health, and they often had to meet with clients in person, despite COVID-19 restrictions, as the client had deteriorated.

To address this concern, service providers identified that a continuum of mental health service delivery, from in person through the web, may be more effective in these populations as clients could choose to receive treatment in person, digitally, or a combination of both. Participants further discussed the possibility that a hybrid model could be used as a way to build rapport between service providers and clients before progressing to a web-based format. Considerations associated specifically with providing trauma therapy using digital health with MMs, veterans, and PSP were also noted.

Managing screen fatigue—the sense of fatigue caused by staring at a computer screen—was also considered critical to the ongoing delivery of mental health services using digital health. Service providers emphasized the importance of self-care, social support, and adequate breaks to do so. It is possible that service providers may need to see fewer clients per day as a result of decreasing billable hours. Fatigue may also be prevalent among clients, necessitating that agencies refrain from overwhelming clients with too much information or on-screen sessions.

The importance of training service providers in the delivery of trauma therapy in a virtual context was emphasized by participants. This may include the use of virtual platforms to deliver therapy and adaptations of therapies to a virtual format, including CPT and eye movement desensitization and reprocessing. Practical issues regarding training were also raised, such as when, where, and how this type of training might be provided.

**Looking forward—service providers.**

**Catalyzing Change**

- *[T]he greatest travesty that could come out of this [...] whole pandemic is to have us revert back to old ways without being able to solidify some of the real values that have come out of it.* [Focus group 3; P21]
- *We have some opportunity right now in this environment to [...] highlight some of these important issues on how we can move them forward.* [Focus group 1; P6]

**Determining Client Suitability for Digital Health**

- *I had to see half of my people in person, because they were [...] deteriorating at home. And so that was sort of an adjustment that I had to make following some of our Provincial recommendations and guidelines from our college.* [Focus group 5; P30]
- *There are lots of treatment programs. [...] [How do] you know which one to use? How do you know if it’s actually being effective in your situation?* [Focus group 4; P24]

**Managing Screen Fatigue**

- *On a really busy clinical day and an exceptional day, I could see eight people in a day and I sort of rolled into the telehealth system with that schedule in place. I’d be wiped out for a couple of days after that. And so I’ve found the busiest day that I can really put in now is about five. And so there is this kind of screen fatigue that happens.* [Focus group 5; P33]

**Providing Continuing Education**

- *Not to say that digital health can’t be used, but as you said, I think it’s a whole other way of being [...] [I]t’s almost as if we’re going to have to retrain all of our clinicians [...] to make sure that people have those skills and those competencies is huge because for me there’s a level of fear and uncomfortableness.* [Focus group 1; P35]
- *I had no virtual care training [...] So that’s a big, big training piece that clinicians should undertake. Now how does that get instituted? How does that get overseen? Who pays for that? This is all time consuming, and clinicians are all so busy to take that on. I think a lot of them are just learning as they go.* [Focus group 1; P3]

##### Organizations

Many organizations and regulatory bodies for regulated health professionals have rapidly developed or enhanced existing policies and practices to support the use of digital health (Textbox 9). Participants noted, however, that policies still require adaptations as the provision of digital health continues to evolve, especially for organizations that had previously been uncomfortable or avoided using digital health. Participants also noted the intentionality required to deliver quality mental health sessions using digital health and strongly suggested eliminating distractions during digital health appointments such as phone calls, emails, and personal interruptions, which were found to be more difficult in a virtual setting than in person. Administrative support and resources were also cited as being essential elements for both clients and service providers in problem-solving technological challenges associated with digital health.

**Looking forward—organizations.**

**Aligning Practice and Policy**

- *I know in our group there’s been such an impetus to move forward that we’re just trying to catch up with policy implementation. We just don’t have the time to get it all done and written and out there. Policy needs to catch up with the process, and that’s a challenge.* [Focus group 1; P3]
- *For organizations, do they now have a policy that supports this sort of virtual online world? And then it’s about ensuring that is the appropriate equipment in place? The technological support if somebody is having a challenge accessing remotely that there’s someone they can call that will help walk them through because there’s nothing more frustrating than a technical problem, when maybe you’re trying to reach out or get support or something like that, right? You just sort of say, well I just give up on that, right.* [Focus group 2; P10]

**Reducing Work Space Distractions**

- *Number one, eliminating distractions. Like, even as I’m sitting here, I’m looking out the window. I’ve been checking my phone. I’ve been plugging away at a draft email. And so it’s harder to eliminate the distractions when we have a venue like this, as opposed to being in the room. So we need to make sure that we’re very clear with folks that we might be doing this for whatever reason, that you need to work extra hard to eliminate the distractions in the room.* [Focus group 4; P22]

**Providing Administrative Supports and Resources**

- *But the idea of having dedicated administrative support to send out the emails, to help clients troubleshoot and feel more comfortable, to walk to a clinician’s office and help them troubleshoot and feel more comfortable. The sooner we can work through some of those barriers, the more comfortable, people will feel because if they continue to struggle, it's easier just to give up and go back to what you know.* [Focus group 3; P15]

##### Clients

Service providers perceived that the use of digital health enhanced service options and reduced barriers for clients accessing mental health services (Textbox 10). They indicated that this was particularly beneficial for clients residing in rural areas where mental health services may be sparse. They also felt that the flexibility of service delivery contributed to a person-centered approach, where a client who may not be ready to access in-person support is still provided service. In addition, in their experiences delivering mental health care using digital health, participants emphasized the need to match generational norms with digital health, with younger generations tending to be more comfortable with digital health delivery.

**Looking forward—clients.**

**Enhancing Service Options**

- *Rural communities or officers that are in two person detachments not having any access to care- like there is no psychologist in a 400 kilometer radius [...] I even look at post-shooting, how many times have we had [...] two officers [...] in a small community and they have nothing, and the communities kind of turn on them. [...] [W]hen we’re looking at smaller communities, this is a game changer for actually having people access what they need.* [Focus group 2; P9]

**Reducing Barriers**

- *[F]or some patients with social anxiety, for example, internet-based sessions are more accessible [...] [I]f they feel demotivated, [...] it’s easier to log in through the internet then it is to come to the clinic.* [Focus group 1; P5]
- *Our goal is always to try to remove all of those barriers. [...] [S]ome people may feel more comfortable starting with text messaging or in person and other people might say the last thing I want to do is actually go in person and talk to somebody, I’m not ready for that yet, but being able to work up. [...] [S]hould we return to a [...] normal world, how do we take the best of what we’re learning and give more options to the people we’re working with? [...] So this pandemic has really forced organizations to pivot really quickly. [...] [and] take a lot more risks than they would have before because they haven’t had any other choices.* [Focus group 2; P10]

**Responding to Generational Differences**

- *I’ve had two members with teenagers that needed to see a clinician, and both member’s kids chose to do it online when they had the option to go in and see the psychologist face to face. [...] [T]hey just felt more comfortable that way. [...] [O]nline’s fine.* [Focus group 2; P13]

## Discussion

### Principal Findings

This study aimed to explore the perspectives of MMs, PSP, and veteran organizational leaders and policymakers and decision-makers; subject matter experts and researchers; and mental health service providers supporting MMs, veterans, and PSP experiencing OSIs amid the transition to digital health because of the COVID-19 pandemic. In light of the impact of the pandemic on health care services and as mental health service provider comfort with and acceptance of technology is a critical component of successful implementation of new initiatives and treatment modalities [29], such an exploration is timely. This study is especially important in that it begins to address the knowledge gap regarding the experiences of those who work within the Canadian context.

Similar themes and a general consensus emerged across focus groups regarding the use of digital health for mental health service delivery to MMs, veterans, and PSP. Almost all participants reported using digital health for mental health service delivery amid the COVID-19 pandemic and suggested that digital health could be adopted as a standard delivery mode for trauma therapy [30]. Similar to research on the use of digital health in the civilian population, some participants identified opportunities and benefits from the widespread adoption of digital health, including more equitable access to mental health services, especially in geographically remote locations; reduction in mental health barriers and stigma; and the ability to develop novel and creative mental health solutions to ongoing challenges [31]. Specifically, as MMs and PSP often work in rural settings and geographically isolated locations, the ability to offer evidence-based mental health treatment to these workers who would otherwise have no access to these supports should command significant attention. Many participants felt that returning to in-person delivery would be a *step back* and that such a return would fail to capitalize on the lessons learned and the work accomplished during the COVID-19 pandemic.

Although digital health provides a number of significant benefits and has proven to be feasible, attention needs to be given to certain issues before its widespread adoption. For instance, systemic changes are needed to effectively facilitate the transition from in-person to digital health service delivery. Of primary concern are the changes needed to update policies and procedures concerning privacy and security. Critical attention needs to be given to platform selection based not only on bottom-line cost but also on functionality and evidence of overall user experience and satisfaction. Such procurement considerations may be at odds with the current legislated policy [32]. Infrastructure, hardware, software, and connectivity at the organizational, clinical, and client levels all need to be explicitly supported. In addition, the workflow, appointments, electronic medical records, liability, and billing codes in place pre–COVID-19 are all based on in-person service delivery and are not always easy to adapt to digital health trauma therapy. Regulatory and reimbursement hurdles faced by individuals and institutions implementing telehealth have been previously published [33,34].

Active support for service providers throughout the transition to the use of digital health for mental health service provision is critical. At the time the focus groups for this study were conducted, participants expressed a degree of openness to digital health delivery. Some mental health service providers participants remained highly skeptical of digital health; however, they further highlighted the critical need for digital health specific training for service providers working with MMs, veterans, and PSP. With the abrupt shift to digital health, mental health service providers found themselves outstripping policies and hastily putting into place ad hoc procedures. They sometimes experienced uncertainty and perceived changes as potentially conflicting with best practices and client interests. Research before the COVID-19 pandemic illustrated that mental health service providers who experienced technological challenges reported feeling more fatigued and experiencing professional self-doubt and a loss of confidence [8,35]. Generational differences may also be an important factor to consider.

Population-specific training would better prepare mental health service providers to deliver quality mental health care to MMs, veterans, and PSP experiencing OSIs. Training is needed to effectively engage with clients when one cannot rely as much on nonverbal cues, as is the case with digital health. The perceived reduction in observed interpersonal cues was frequently identified as a challenge by mental health service providers in Britain using digital health during COVID-19 [31]. As nonverbal cues are a key component of trauma therapy, the reduction or removal of cues represents a potential loss of valuable information in the treatment process [31]. This is particularly significant when delivering trauma therapy to clients with complex mental health needs and at an increased risk of harm to self or others. Such concerns become even more salient when clients are highly trained professionals who have knowledge of weaponry and lethal force, which can put them at greater risk for completed suicide.

Mental health service providers were acutely aware of the need to be attentive to subtle verbal and nonverbal cues to establish the therapeutic relationship, deliver trauma therapies, and assess both treatment effectiveness and risk of self-harm. Geller [33] had argued that when mental health service providers are “fully in the moment” and attuned with their clients, this sense of mutual safety and strong alliance invites clients to engage in the necessary therapeutic work by increasing their ability to emotionally regulate. Although not fully known at this time, this attunement and subsequent emotional regulation may be lessened in the context of digital health [36]. Research into *Zoom fatigue* indicates that videoconferencing is more mentally taxing, the level of communication is not completely synchronous, and greater cognitive energy is required [35,37]. Cognitive demand may be intensified by the lack of nonverbal cues, which would typically assist communication during in-person encounters [37]. Within the context of intensive trauma therapy, the cognitive endurance required may be even greater than what would be required for an in-person therapeutic encounter.

The appropriateness of digital health for clients is also dependent on cognitive functioning, the source and type of trauma, and technical literacy. Given that cognitive impairment (including memory, attention, concentration, and executive processes) is fairly common in trauma-induced mental health disorders, it is unclear whether some elements of digital health could be specifically problematic or challenging to certain MMs, veterans, and PSP [38,39]. Jones et al [19] determined that the use of digital health for trauma therapy in these populations showed comparable results to that of in-person therapy. Similar results have been found regarding the feasibility of telehealth for serious mental illnesses (ie, schizophrenia, schizoaffective disorder, and bipolar disorder) [37,38]. Such evidence suggests that digital health, or at least the use of a hybrid approach (blend of digital health and in-person services), could be effective for MMs, veterans, and PSP. It is possible that some clients may benefit from in-person delivery as rapport is developed, with the delivery of therapy shifting to digital health platforms once the therapeutic alliance is well established. There may also be certain aspects of rehabilitation and recovery that may be more or less suited to digital health. For example, clients who require acute stabilization may be better suited for in-person service delivery and monitoring compared to those with more stable symptoms. A continuum of service offerings and flexible and adaptable delivery mechanisms on the part of mental health service providers may best meet the needs of MMs, veterans, and PSP experiencing OSIs and seeking mental health treatment. Delivering safe, effective, and timely trauma therapy to MMs, veterans, and PSP by mental health service providers who are comfortable with both in-person and digital health environments would significantly broaden service delivery options and likely result in increased access to mental health care.

### Recommendations and Future Research

As digital health is a novel method for delivering trauma therapy, it requires further systematic research and should not currently be considered synonymous with in-person therapy. The aforementioned considerations should be explored, and modifications should be made to ensure safe and effective mental health care to widely implement digital health delivery of mental health services. Research into digital health is imperative to define the best approaches for effective digital health-delivered care and treatment of these and other patient populations. Special consideration should be given to the fit of technology within the organization, environment, culture, and other relevant contexts [39].

Key recommendations from this study:

- Specific infrastructure, technologies, policies, and procedures are needed if digital health is to be permanently integrated as a predominant mode of mental health service delivery.
- Mental health service providers need access to administrative, technological, colleague support, and recognition and mitigation of new job demands in a digital health environment.
- Training is required if mental health service providers are to effectively, comfortably, and competently deliver trauma therapies to MMs, veterans, and PSP in a digital health environment.
- A hybrid model may bridge services from in-person to digital health service provision and allow for the delivery of flexible, personalized care.
- Proactive outreach is needed to support clients with OSIs.
- Clients also need to be educated about protocols, procedures, potential benefits, and challenges associated with digital health service delivery.

In addition, if digital health is to be broadly used as a medium for mental health delivery for MMs, veterans, and PSP, further research is needed in a number of areas. First, further exploration regarding which trauma therapies are most conducive to digital health environments is required. To date, most of the research on the delivery of digital health trauma interventions has focused on CPT and prolonged exposure, with limited studies on eye movement desensitization and reprocessing [19]. Research is also needed to determine the indications and contraindications to the use of digital health. Significant research is needed regarding the management and mitigation of risk when delivering digital health trauma therapy. Finally, research should explore the potential of digital health to be used to deliver novel and creative trauma therapy. In this study, some participants highlighted the possibility of delivering therapy when a client was walking, whereas others noted that trauma therapy could be provided via smartphones.

### Limitations

This study had some limitations. First, this study used a small convenience sample, which drew on pre-existing relationships with national, provincial, and municipal military, veteran, and PSP partners. Second, the ability to recruit was affected by the COVID-19 pandemic as two of the populations of this study (ie, active-duty MMs and PSP) were frontline SPs during the pandemic and significant work demands limited their ability to engage in the study. Third, there were limited frontline participants (potentially because of the impact of the pandemic on their work), resulting in more perspectives being gathered from policy makers and SPs than from persons with lived experience. Consequently, the views of client experiences described in this study are mostly from the perspective of clinicians or mental health service providers. Finally, data may have been biased by focus group participants being potentially swayed by the views of others or participants not feeling comfortable disclosing their personal views in a group.

### Conclusions

This study explored the use of digital health for the provision of mental health services during the COVID-19 pandemic from the perspective of stakeholders, including mental health service providers and policy makers, providing trauma therapy to MMs, veterans, and PSP. The findings suggest that digital health is a viable component of a model of care for MMs, veterans, and PSP, which is inclusive of in-person and virtual modes of trauma-focused service delivery. The findings offer considerations for whom and at what point in treatment digital health is appropriate; clarification of training, support, resources, and guidelines necessary for service providers to be successful in the digital delivery of trauma therapy; and a better understanding of the factors influencing service provider perceptions and acceptance of digital health. These results can inform the implementation and uptake of mental health interventions via digital health for MMs, veterans, and PSP and may equally apply to other trauma-affected populations. As the COVID-19 pandemic continues, remote service delivery methods for trauma therapy will be increasingly needed to support the mental health and well-being of MMs, veterans, and PSP who continue to serve and respond to the needs of communities.

## Appendix

Multimedia Appendix 1 Focus group (World Cafe) or interview questions.

## Abbreviations

CPT: cognitive processing therapy
MM: military member
OSI: operational stress injury
PSP: public safety personnel
REDCap: Research Electronic Data Capture
