# Supplementary material for: The Experience of Key Stakeholders During the Implementation and Use of Trauma Therapy via Digital Health for Military, Veteran, and Public Safety Personnel: Qualitative Thematic Analysis
Source: JMIR Form Res. 2021 Aug 12;5(8):e26369. doi: 10.2196/26369 (PMC8391743; doi:10.2196/26369)
Supplement: Multimedia Appendix 1 [file formative_v5i8e26369_app1.docx]

Multimedia Appendix 1: Focus Group (World Cafe) or Interview Questions

1. **Current State and Needs Analysis:**

a. What has the experience been like regarding the rapid transition to remote digital

health (DH) mental health care?

b. Broadly speaking and in a perfect situation, what would the use of DH look like

and why?

c. Were there any unexpected outcomes? Positive or Negative?

2. **Barriers / Supports / Facilitators**

a. What parts of the transition to DH was most challenging and why?

b. What parts of the transition to DH were the easiest and why?

3. **Technological Issues**

a. Have there been any logistic or security issues (i.e., firewalls, compatibility, or

policies) that have challenged the ability to use DH?

b. What has the feasibility been like?

4. **Technological Acceptance** - What has the technological acceptance been like? What

has the feedback been like from clinicians? Frontline personnel? Care recipients?

Persons with lived experience? Care givers? IT specialists?

5. **Methods of Delivery** – What would be the best way to deliver mental health for PTSIs?

Telehealth? Videoconferencing? Apps?

6. **Clinical Effectiveness:**

a. Has use of DH for PTSI therapy been effective from the perspective of persons

with lived experience? Interdisciplinary mental health clinicians? Leaders? Care

givers? Describe

b. Were you satisfied with the care? Explain.

c. Were you satisfied with the infrastructure? Explain.

7. **Implementation Needs** - What would need to change to better integrate DH in practice?

a. What will/should stay the same/evolve?

b. What would successful implementation look like and why?

c. What - What steps / practices would enable the use of DH to be brought to life?

(practices, approaches, activities, resources, etc.)

d. Who - should help mobilize use of DH?

e. Scaling and sustainability – is the ongoing use of DH sustainable? Ought the

use of DH continue after COVID-19? Describe and explain

f. What infrastructure – is needed to disseminate knowledge?

8. **Knowledge Dissemination** - What knowledge do you need to be able to decide if DH is

appropriate for use? How would this information best be given to you? What do you wish

you had known prior to COVID-19 and the transition to DH use?
